# Circadian and chemotherapy-related changes in urinary modified nucleosides excretion in patients with metastatic colorectal cancer

**DOI:** 10.1038/s41598-021-03247-2

**Published:** 2021-12-14

**Authors:** S. Dulong, Q. Huang, P. F. Innominato, A. Karaboue, M. Bouchahda, A. Pruvost, F. Théodoro, L. A. Agrofoglio, R. Adam, B. Finkenstädt, F. Lévi

**Affiliations:** 1 Chronotherapy, Cancer and Transplantation, University of Paris Saclay, Faculty of Médecine, Bat A 3rd Floor, Campus CNRS, 7 rue Guy Moquet, 94800 Villejuif, France; 2 University of Paris Saclay Faculty of Medecine, Le Kremlin Bicetre, France; 3 Cancer Chronotherapy Team, Cancer Research Centre, Warwick Medical School, Gibbet Hill Road, Coventry, CV4 7AL UK; 4 Department of Statistics, University of Warwick, Coventry, CV4 7AL UK; 5 North Wales Cancer Centre, Ysbyty Gwynedd, Betsi Cadwaladr University Health Board, Bangor, UK; 6 Department of Oncology, Paul Brousse Hospital, Assistance Publique-Hôpitaux de Paris, Villejuif, France; 7 Mousseau Clinics, Ramsay Générale de Santé, Evry, France; 8 CEA, INRAE, Département Médicaments et Technologies pour la Santé (DMTS), SPI, Université Paris-Saclay, 91191 Gif-sur-Yvette, France; 9 ICOA, UMR7311, CNRS, University of Orleans, Orleans, France; 10 Department of Chirurgie Centre Hépato-biliaire, Paul Brousse Hospital, Assistance Publique-Hôpitaux de Paris, Villejuif, France

**Keywords:** Colon cancer, Prognostic markers

## Abstract

Urinary levels of modified nucleosides reflect nucleic acids turnover and can serve as non-invasive biomarkers for monitoring tumour circadian dynamics, and treatment responses in patients with metastatic colorectal cancer. In 39 patients, median overnight urinary excretion of LC-HRMS determinations of pseudouridine, was ~ tenfold as large as those of 1-methylguanosine, 1-methyladenosine, or 4-acetylcytidine, and ~ 100-fold as large as those of adenosine and cytidine. An increase in any nucleoside excretion after chemotherapy anticipated plasma carcinoembryonic antigen progression 1–2 months later and was associated with poor survival. Ten fractionated urines were collected over 2-days in 29 patients. The median value of the rhythm-adjusted mean of urinary nucleoside excretion varied from 64.3 for pseudouridine down to 0.61 for cytidine. The rhythm amplitudes relative to the 24-h mean of 6 nucleoside excretions were associated with rest duration, supporting a tight link between nucleosides turnover and the rest-activity rhythm. Moreover, the amplitude of the 1-methylguanosine rhythm was correlated with the rest-activity dichotomy index, a significant predictor of survival outcome in prior studies. In conclusion, urinary excretion dynamics of modified nucleosides appeared useful for the characterization of the circadian control of cellular proliferation and for tracking early responses to treatments in colorectal cancer patients.

## Introduction

Colorectal cancer ranks as the second cause of cancer-related deaths, which makes it critical to detect and treat this malignancy as early as possible in order to enhance survival and cures. Elevated circulating levels in carcinoembryonic antigen (CEA) and carbohydrate antigen CA19.9 have proven their clinical usefulness for assessing treatment responses and detecting early relapse^1^. More recently, circulating tumor cells, cell-free DNA and RNA or exosomes, have been proposed as blood biomarkers for monitoring colorectal malignancies^2^. The several nucleosides that result from DNA or RNA breakdown undergo post-transcriptional chemical modifications yielding ultrafilterable modified nucleosides that cannot be re-utilized or be degraded further, and are excreted as such into the urines. These modified nucleosides belong to the family of the ribosyl-nucleosides, twenty of which have been proposed as tumor markers for cancers of the colon or liver^3,4^, breast^5–7^ or lung^8^. More specifically, the isomerisation of uridine, a nucleic acid base that composes RNA, results in pseudouridine, which is incorpored into tRNA and released in the systemic circulation then ultrafiltrated into the urine following nucleic acids breakdown, primarily including tRNA^9^. The desamination of adenosine yields inosine. The methylation of common bases that compose DNA or RNA lead to the formation of 1-methyladenosine, 1-methylinosine, 1-methylguanosine, and N2-N2-dimethylguanosine. Modified DNA base 4-acetylcytidine results from the desacetylation of cytidine. All these biochemical reactions occur at post-transcriptionnal level, and have been reported as being enhanced in cancer, as a result of increased cellular metabolism and accelerated RNA turnover in tumor cells^10,11^.

The current study aimed to determine whether these modified nucleosides could serve as non-invasive and quantifiable markers of dynamic changes in tumors, along the 24-h and/or after chemotherapy. Indeed, 24-h changes have been documented for several circulating tumour biomarkers, including carcinoembryonic antigen (CEA) and CA125^12–14^, as well as for urinary excretion patterns of polyamines or myelomatous immunoglobulin^15^. While rhythms were scarcely detectable in groups of patients, strikingly rhythmic patterns were demonstrated in individual patients^12,14^. The identification of such patient-specific circadian control of cancer processes could in turn help optimize treatment timing and enhance efficacy.

The circadian timing system (CTS) is a hierarchically coordinated network of molecular clocks that regulates mammalian physiology, as well as cellular metabolism, proliferation, and survival along the 24-h^16,17^. The disruption of the rhythms that are generated by the CTS has been associated to disease processes and impaired treatment effects (for review see^18–20^). Circadian rhythms are generated at single-cell level by molecular clocks, consisting of interwoven transcriptional translational feedback loops involving some 15 known specific “*clock*” genes including Bmal1, Clock, Period 1 and 2, Cryptochrome 1 and 2 and Rev-erbα^21,22^. The molecular clocks are coordinated at the whole organism level by the suprachiasmatic nuclei, a hypothalamic pacemaker, which also helps circadian rhythms adjust to light–dark and other environmental 24-h cycles through the rhythmic control of rest-activity, body temperature, feeding, as well as cortisol and melatonin secretions and autonomic nervous sytem activities^1^. Both glucocorticoids and body temperature rhythms reset molecular clocks and cellular circadian rhythms both in vitro and in vivo^23–27^.

The evaluation of CTS robustness or disruption in healthy or sick humans during their daily routine has mostly involved the non-invasive recording of the rest-activity rhythm, using a wrist-worn accelerometer. In cancer patients, the relative amount of activity in bed that was below the median activity out of bed, the so called dichotomy index I < O, has been identified as an independent predictor of progression-free survival and overall survival among 436 patients with metastatic colorectal cancer^28,29^.

Here we determined the urinary excretions of up to 8 nucleosides in patients with metastatic colorectal cancer recruited in two studies. We assessed inter- and intra-patient changes in these potential biomarkers along the 24 h (St1), or before, during and after chronochemotherapy (St2). We linked the 24-h variations in nucleosides excretion with the rest-activity circadian rhythm and characterized the potential clinical significance of their dynamics.

## Results

### Patients characteristics

**Figure 1:**
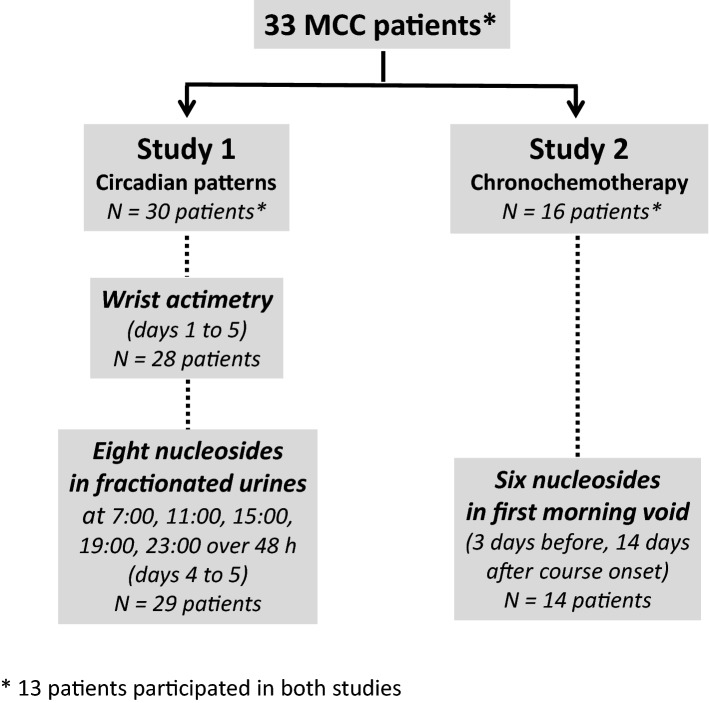
Consort diagram of both studies in patients with metastatic colorectal cancer (MCC). Thirteen patients participated in both studies.

Both observational studies (St) were conducted in a total of 33 patients with metastatic colorectal cancer. Thirteen patients participated to both studies at various stages of their disease (Fig. 1, Table 1). St1 recruited 30 patients, the majority of whom had a WHO performance status of 0 (N = 23, 76.7%), primary colon cancer (N = 66.7%), and liver metastases (N = 20, 86.7%). Thirteen patients had two or more metastatic sites (43%). All but one patient had received prior chemotherapy, with 2 prior protocol lines being administered for 93.3% of the patients. Sixteen patients (53.8%) had undergone prior surgery of metastases. Five-days wrist watch monitoring was complete for 28 out of 30 patients (93.3%). There were two technical failures, with no data collection. Two hundred and ninety-three urine samples were collected. The number of samples was insufficient for a single patient. Overall, a total of 226 nucleoside excretion data points were available for analysis in 29 patients, corresponding to a protocol compliance rate of 95.9%. St2 involved 16 patients who received a chronochemotherapy protocol. The majority of the patiens had colon cancer (N = 11, 69%) with liver metastases (N = 12, 75%). 94% of them had prior resection of the primary tumor (N = 15), and 63% of them also had prior metastases surgery (N = 10). The majority of the patients (N = 14, 88%) had received more than 2 lines of chemotherapy. A total of 239 urinary samples were analysed. Overall, nucleosides excretions were determined in 465 urinary samples in both studies.

**Table 1:** Main characteristics of the patients in both studies (St).

	St1	St2
Number of patients	30	16
**Sex**		
Male	18	8
Female	12	8
**Age, years**		
Median	66.2	67.5
Range	23–74	44–81
**Performance status (WHO)**		
0	23	12
1	7	4
**Primary site**		
Colon	20	11
Rectum	10	5
**Metastatic site**		
Liver	22	12
Lung	10	8
Others	18	10
**Number of metastatic sites**		
1	17	6
2	6	3
3	7	7
**Comorbidities**		
Yes	14	10
No	16	6
**Prior primary tumor surgery**		
Yes	27	15
No	3	1
**Prior metastatic surgery**		
Yes	16	10
No	14	6
**Number of prior chemotherapy lines**		
0	1	0
1	1	2
2–3	22	11
More than 3	6	3
**Prior chemotherapy modality**		
Chronotherapy	23	12
Conventional	7	4
**Plasma CEA (mg/L)**		
< 5	6	3
6–100	17	10
> 100	4	2
Unknown	3	1

### Overnight nucleosides excretions in the absence of treatment

**Figure 2:**
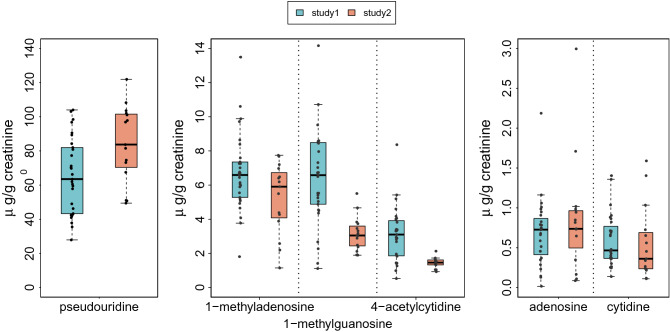
Distribution of early morning urinary excretion values of the six modified nucleosides at baseline in both studies. Nucleosides excretions are ranked from highest (pseudouridine) to lowest (cytidine). Median values, interquartile and extreme values are shown as boxplots for each nucleoside in each study. Slight differences in median values and/or range of pseudouridine, 1-methylguanosine and 4-acetylcytidine excretions between patients in St 1 and patients in St 2.

Within each St, the early morning levels of urinary nucleoside excretion, corresponding to overnight elimination into the urine, varied by up to nearly 100-fold according to nucleoside type in the absence of any treatment for the past 2 weeks (Fig. 2). The excretion of pseudouridine was nearly tenfold as large as those of 1-methylguanosine, 1-methyladenosine and N2-N2-dimethylguanosine, and 20–30-fold as large as those of 4-acetylcytidine and 1-methylinosine. The nucleosides with lowest excretions were adenosine and cytidine. Interpatient variability was large with coefficients of variation ranging from 84.2% for adenosine in St 1 to 21.8% for 4-acetylcytidine in St 2, with a median value of 46.5% [IQR, 33.9 to 60.9%] across nucleosides and studies. Two-sample *t*-test revealed that the overnight excretion of pseudourine was higher in patients with co-morbidities (p < 0.001), that of 1-methyladenosine was higher in female as compared to male patients (p = 0.039), and that of adenosine was higher in those patients with primary colon rather than rectum cancer (p = 0.015).

### Twenty-four hour changes in urinary nucleosides excretions

In St 1, detectable urinary levels were found in all the patients for eight nucleosides, but not for 8-hydroxy-2′-deoxyguanosine.

#### Average 24-h excretions (mesors)

The median level of urinary nucleoside excretion mesors varied by up to 110-fold according to nucleoside type among the 29 patients (Fig. 3.1). Thus, the excretion of pseudouridine (median, 64.3 µg/g of creatinine) was nearly tenfold as large as those of 1-methylguanosine, 1-methyladenosine and N2-N2-dimethylguanosine, and 20–30-fold as large as those of 4-acetylcytidine and 1-methylinosine. The nucleosides with lowest excretions were adenosine and cytidine (medians of 0.69 and 0.61 µg/g of creatinine respectively). Large inter-patient variations were demonstrated for the urinary excretion of the modified nucleosides, with coefficients of variation ranging from 25.0% (pseudouridine) to 42.3% (1-methylguanosine).

**Figure 3:**
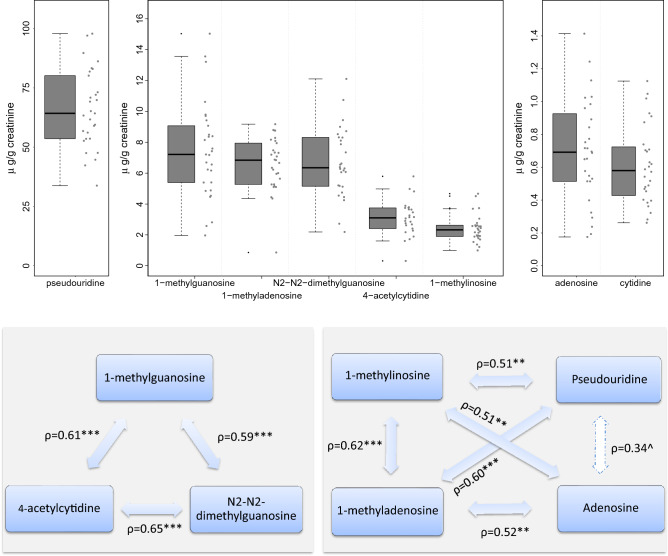
Distribution of urinary excretion mesor values for the 8 modified nucleosides and their intercorrelations in St 1. (1) Mesor estimates with the boxplots (median, interquartile and extreme values) for each nucleoside, ranked from highest to lowest median excretion values. (2) Diagram of intercorrelations. Spearman’s rank correlation coefficients ρ with stars indicating p-value levels, i.e. 0 ‘***’ 0.001 ‘**’ 0.01 ‘*’ 0.05 ‘^’ 0.1. Insignificant correlation in bootstrap uncertainty check, i.e. pseudouridine and adenosine, is marked with dashed line.

The mesors of nucleosides excretion were correlated among patients, except for cytidine. More specifically, two clearly distinct groups of inter-correlated nucleosides were identified (Fig. 3.2). A first one, involving pseudouridine, 1-methyladenosine, 1-methylinosine, and adenosine, and a second one involving 1-methylguanosine, N2-N2-dimethylguanosine and 4-acetylcytidine.

#### Twenty-four-hour patterns

**Figure 4:**
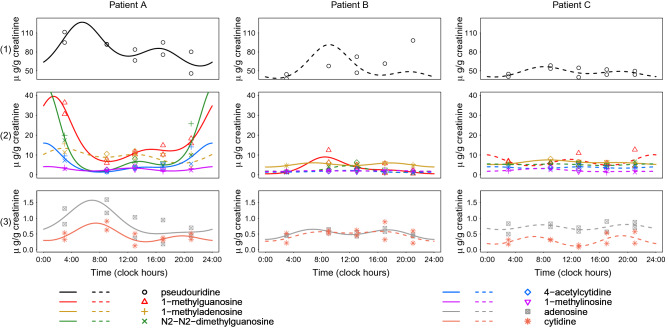
Examples illustrating interpatient differences in 24-h excretion patterns of urinary nucleosides in St 1. Urinary nucleoside excretion measurements (dots) and multiple-component cosinor model fitting (lines) for example patients A, B and C. Nucleosides identified with significant circadian rhythmic pattern are plotted in solid line, while non-significantly rhythmic ones are shown in dashed line. Nucleoside patterns from each patient are plotted in three panels with different ordinate scales.

Using multiple-component cosinor analysis, the number of nucleoside excretion rhythms per patient ranged from null to 7, with a median of 3. Raw nucleosides measurements and cosinor model fitting are shown as examples for three patients (A, B and C) (Fig. 4). Compared with the relatively flat fitting curves for patient B and C, patient A displayed a large circadian variability in nucleosides excretion and consistent day-to-day patterns. As a result, patient A had rhythmic excretions for 7 nucleosides, with significantly reproducible 24-h patterns (plotted with solid lines), while patient B had 3 excretion rhythms and patient C a single one.

Overall, the urinary excretion of each nucleoside displayed a 24-h rhythm for 4-acetylcytidine in 6 of 29 patients (23.1%), and for pseudouridine in 11 of 29 patients (37.9%). A 12-h rhythm was also found 7 of 29 patients (24.1%) for pseudouridine and for both 1-methylguanosine and 1methyladenosine in 5 of 29 patients (17.2%). Thus a 24-h and/or a 12-h rhythm characterized pseudouridine urinary excretion for 14 of 29 patients (48.3%) of the patients (highest rate) as compared to 4-acetylcytidine for 10 of 29 patients (34.6%) of the patients (lowest rate).

Statistically significant correlations were found for the urinary excretion rhythm amplitudes as % of mesor for 1-methylinosine (Spearman’s correlation with p-value = 0.001), 1-methylguanosine (p < 0.001), 1-methyladenosine (p = 0.004), N2-N2-dimethylguanosine (p < 0.001), cytidine (p < 0.001) and 4-acetylcytidine (p = 0.017). Circadian mesor, amplitude and acrophase estimates for the eight nucleosides excretions are summarised in Table 2.

**Table 2:** Results from cosinor analyses of nucleosides urinary excretion in St 1.

	1-methylinosine	pseudouridine	1-methylguanosine	1-methyladenosine	N2-N2-dimethylguanosine	adenosine	cytidine	4-acetylcytidine
Number of patients	29	29	29	29	28	28	28	26
Number of patients with significant rhythmicity pattern	7	11	10	10	9	9	10	6
Mesor (µg/g creatinine)	2.41 (0.99–4.67)	66.1 (33.8–97.9)	7.44 (1.97–15.04)	6.57 (0.86–9.18)	6.70 (2.19–12.11)	0.69 (0.18–1.41)	0.61 (0.26–1.13)	3.09 (0.31–5.80)
	2.32 [1.90, 2.63]	64.3 [53.6, 80.2]	7.22 [1.97, 9.08]	6.84 [5.27, 7.95]	6.35 [5.16, 8.31]	0.69 [0.51, 0.91]	0.58 [0.44, 0.72]	3.11 [2.46, 3.73]
Amplitude (µg/g creatinine)	0.98 (0.21–2.95)	26.9 (5.74–60.7)	5.57 (1.02–16.36)	1.94 (0.66–4.69)	5.63 (0.38–21.48)	0.39 (0.08–1.24)	0.36 (0.13–0.81)	2.28 (0.29–7.30)
	0.71 [0.46, 1.31]	26.7 [16.3, 34.7]	5.24 [3.21, 7.28]	1.76 [0.66, 4.69]	4.74 [2.75, 6.55]	0.24 [0.16, 0.53]	0.28 [0.20, 0.52]	1.49 [0.85, 3.18]
Relative amplitude (% mesor)	0.40 (0.09–1.26)	0.42 (0.06–0.99)	0.80 (0.17–2.07)	0.31 (0.15–0.76)	0.79 (0.07–2.59)	0.69 (0.12–3.11)	0.59 (0.24–1.07)	0.73 (0.12–2.51)
	0.33 [0.23, 0.48]	0.41 0.25 0.54]	0.72 [0.59, 1.00]	0.28 [0.20, 0.38]	0.71 [0.49, 1.01]	0.41 [0.31, 0.68]	0.58 [0.43, 0.72]	0.66 [0.32, 0.95]
Acrophase (clock hours and min.)	08:26 (00:06–23:18)	13:06 (01:30–23:42)	13:00 (00:06–23:42)	10:08 (00:01–23:36)	11:13 (00:00–23:30)	10:44 (00:24–23:36)	11:39 (00:06–23:48)	13:30 (00:06–23:54)
	06:42 [02:36, 12:24]	12:54 [09:06, 19:00]	12:00 [7:42, 19:18]	9:48 [04:18, 14:42]	10:24 [05:88, 19:01]	10:27 [05:42, 12:48]	12:27 [05:20, 17:50]	13:06 [06:45, 20:48]

For each parameter, i.e. Mesor, Amplitude, Relative amplitude and Acrophase, mean values and (ranges) are reported in upper rows, and median values and [1st, 3rd] quartiles are given in lower rows, using imputed rhythm parameter values from each individual patient.

### Circadian patterns in rest-activity

**Figure 5:**
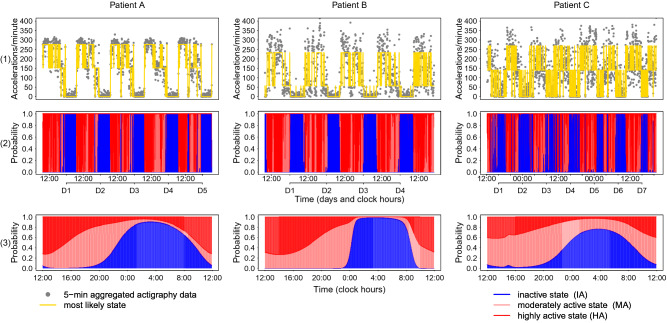
Interpatient differences in 24-h patterns in rest-activity. Profiles of the same three patients in St 1 that were selected for Fig. 4. (1) Plotted time series of wrist watch activity measurements. Time series of activity in dots, and corresponding HMM-decoded most likely states connected with a yellow line (lowest level for IA—inactive, middle level for MA—moderately active, and highest level for HA—highly active). (2) Corresponding most likely states, i.e. IA, MA or HA, with frequent state transitions between MA and HA during daytime. Cumulative state probability plots for IA (blue), MA (light red) and HA (dark red) states. (3) Temporal distribution of the three state probabilities over the 24 h, providing a fingerprint of the individual’s typical daily rest-activity behaviour. Patients A and B had solid blue areas (IA) at night while patient C had many interruptions. This was translated into low values for p1-1 and I < O. A sharp incline/decline of the blue IA profile is indicative of regular times for retiring in the evening and awakening in the morning. The prolonged IA period of Patient B started around 0:00–1:00 a.m. and ended around 8:00–9:00 a.m. every day with no noticeable interruptions to the IA state in-between, hence this patient has the highest Rhythm Index among the three patients shown. Values of rest-activity indices: patient A: I < O: 99.7%, Rhythm Index = 67.3%, p1-1 = 0.969, r24 = 0.654; patient B: I < O: 98.2%, Rhythm Index = 91.2%, p1-1 = 0.972, r24 = 0.392; patient C: I < O: 90.6%, Rhythm Index = 54.6%, p1-1 = 0.910, r24 = 0.247.

The 24-h pattern in rest-activity of the 28 patients in St 1 ranged from robust to weak as indicated by the results from Hidden Markov modelling, dichotomy index I < O, autocorrelation coefficient r24, circadian period estimates and 24-h cosinor estimates of amplitude and acrophase (Table S1). Interestingly, the median I < O value was 97.9%, in good agreement with prior studies on larger sample sizes, where this value was 97.5%^28^. Interpatient differences in rest-activity patterns were obvious (Fig. 5). The timeseries of wrist-watch activity measurements, the corresponding displays resulting from HMM, and the temporal distribution of High, Intermediate and Low Activities over the 24 h are shown in Fig. 5, for the same three patients as those taken as illustrative examples for nucleosides rhythmic excretions (Fig. 4). Patient B had a regular and robust rest-activity rhythm, patient C had a severely disturbed pattern and patient A displayed an intermediate profile.

The dichotomy index I < O, the Rhythm Index, the transition probability of remaining in IA state (p1-1), and the autocorrelation coefficient at 24-h (r24) were strongly correlated with each other, as the Spearman rank coefficients $\rho$ ranged from 0.49 (p = 0.009) between I < O and the Rhythm Index, up to 0.80 (p < 0.001) between I < O and p1-1. The median level of the HA state was also correlated with both I < O ($\rho$ = 0.40 with p = 0.033) and r24 ($\rho$ = 0.58 with p = 0.001). The overall daily rest duration was negatively correlated with the Rhythm Index ($\rho$ = − 0.49 with p = 0.007) (Fig. S1).

Multivariate regression analysis revealed that a good performance status (PS = 0) was significantly associated with high values in I < O (p = 0.007), p1-1 (p < 0.001), and Rhythm Index (p = 0.007). Female sex was associated with high values in p1-1 (p = 0.024) and median activity at HA state (p = 0.011), and possibly with high values in both I < O and r24 (0.05 $\le$ p $<$ 0.1).

### Relations of nucleosides excretion changes with patient characteristics and rest-activity pattern

Multivariate regression analysis showed that the mesor of 1-methylguanosine excretion was higher in patients with a performance status of 1 as compared to those whose PS was 0 (p = 0.026). The mesor of pseudouridine and that of adenosine were lower in the patients who had prior liver metastases surgery (p = 0.049 and p = 0.007, respectively). The circadian amplitudes of adenosine and N4-acetylcytidine were higher in female as compared to male patients (p = 0.011 and p = 0.01, respectively).

**Figure 6:**
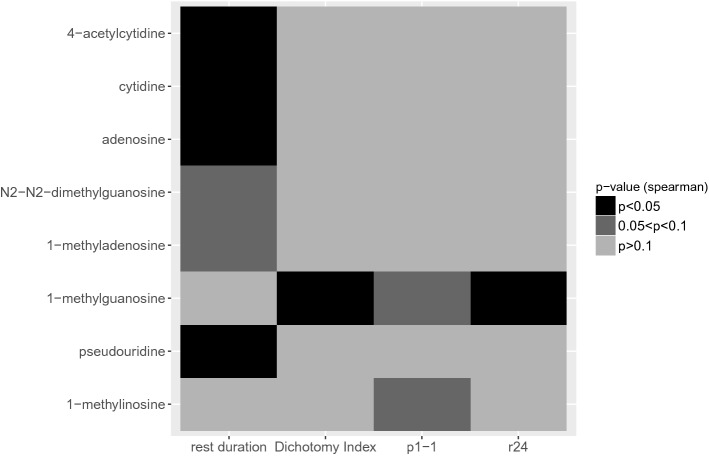
Grid plots of intercorrelations between selected rest-activity indices and urinary nucleoside excretion relative amplitudes in St 1. Black boxes correspond to statistically significant positive (+) or negative (−) Spearman’s correlations (p $\le$ 0.05). Dark grey boxes correspond to trends (0.05 $<$ p $\le$ 0.1), and light grey boxes correspond non significant correlations (p $>$ 0.1).

Spearman’s rank correlation analysis revealed striking relations between the relative circadian amplitude of the 24-h patterns of urinary nucleosides excretions and rest-activity indices (Fig. 6). For example, the larger the rest duration, the larger the relative circadian amplitude of pseudouridine (p = 0.015), adenosine (p = 0.049), 4-acetylcytidine (p = 0.008) and cytidine (p = 0.002). Moreover, the more robust the rest activity rhythm, as indicated with a higher I < O, the higher the relative circadian amplitude of 1-methylguanosine (p = 0.0182).

### Changes after chronochemotherapy, and relations with outcomes

No urinary excretion data were available for one patient in St2. Chronochemotherapy administration was followed with increasing or decreasing trends in overnight urinary nucleosides excretions in six patients or no change in the other nine patients (see four illustrative examples (one with positive, one with negative, two with no trend for cytidine in Fig. S2). The data thus highlighted patient-specific treatment responses and dynamics, which were recapitulated by the changes in 1-methylguanosine, acetylcytidine, adenosine and cytidine**.** Having an increasing or a decreasing trend (slope of regression line with p-value $\le$ 0.05) in these urinary nucleosides after chemotherapy was associated with a > 50% delayed increase in plasma CEA (Fisher's exact test p-value = 0.0275) and a poor survival (two-sample t-test, p-value = 0.0054) (Table 3). In contrast, no change (p > 0.05) in nucleosides was associated with no change in plasma CEA, no toxicity > Grade 1, and a far better survival in this limited sample of metastatic colorectal cancer patients.

**Table 3:** Relations between chemotherapy-related changes in urinary nucleosides and those in plasma carcinoembryonic antigen (CEA) for the patients in St 2.

Trend in nucleosides excretion	CEA			
	Decrease	No change	Increase	Sum
No trend	1	8	0	9
Trend	0	2	3	5
Sum	1	10	3	14

Fourteen patients with both nucleosides and CEA determinations are considered . Changes in plasma CEA are categorized according to whether this tumor marker increased or decreased by more than 50% (trend) or remained stable for at least 1 month and up to 2 months after a course of chronochemotherapy. Changes in nucleosides are classified as trend, in case of a positive or a negative slope of regression line (p-value $\le$ 0.05), respectively or as no change in case of no slope (p-value $>$ 0.05). The interaction between nucleoside excretion trend and CEA change was evaluated by Fisher chart, p-value = 0.0275.

## Discussion

In patients with previously treated metastatic colorectal cancer, the average overnight urinary excretions of six modified nucleosides varied by over 100-fold according to the nucleoside considered, being highest for pseudouridine, intermediate for 1-methyladenosine, 1-methylguanosine and 4-acetylcytidine, and lowest for cytidine and adenosine. Large differences were found between patients before liver surgery or chronochemotherapy, as well as along the 24-h time scale, that partly related to sex, co-morbidities, and primary colon or rectum site.

Consistently with our findings, large between- and within-patients variations were reported for pseudouridine, 1-methyladenosine and 1-methylguanosine in patients with breast, lung, or gastric cancer. In these patients however cytidine and adenosine urinary excretions were larger, and 4-acetylcytidine was only occasionally determined^10,11,30^. In metastatic colorectal cancer patients, we found striking similarities between the urinary excretions of all the nucleosides in both studies. As compared to results in other cancer studies mentioned in our manuscript, we found similar levels for pseudouridine (~ 100 µg/g of creatinine), N2-N2-dimethylguanosine (~ 6 µg/g of creatinine) and 1-methylguanosine (between 0.43 and 9.3 µg/g of creatinine, pending on the studies , i.e. 6 or 3 µg/g of creatinine respectively). In contrast, large between-studies variabilities stood out for the other urinary nucleosides excretion that possible related to differences in determination techniques, cancer types, and/or stage or grade. Thus, mean urinary excretions ranged from 6.5 µg/g of creatinine (our studies 1 and 2) up to 42 µg/g of creatinine for 1-methyladenosine^11^, and from 2 µg/g of creatinine (our studies) up to 36 µg/g of creatinine for 1-methyl1inosine^11^. Moreover, our mean adenosine and cytosine excretion levels were 20 times and 6 times as low as those reported for earlier stage colorectal cancer patients^30,31^.

In our study, chronochemotherapy resulted in patient-specific responses in the overnight urinary excretions of the six modified nucleosides. Increasing, stable, or decreasing trends were captured in the urinary excretions of 1-methylguanosine, 1-methyladenosine, adenosine and cytidine. Consistent increases in urinary nucleosides excretions were related to disease progression on chronochemotherapy (St2). These observations suggest that these four urinary nucleosides levels could reflect cellular proliferation and serve as early markers of efficacy of chemotherapy.

The 24-h mesors of urinary excretion levels of the modified nucleosides in St1 ranked similarly as the overnight excretions that were determined in St2. Eight-hydroxy-2-deoxyguanosine, the ninth modified nucleoside selected for St1, was not detected in 68% of the diluted urine samples. A circadian rhythm in the urinary excretion of this oxidized form of guanosine had been previously reported both in healthy adults and in patients with multiple sclerosis, but not in diabetic patients^32,33^. Moreover, elevated levels of 8-hydroxy-2-deoxyguanosine were reported in cancer patients^34,35^. Here, owing to the dilution of the samples before injection, this modified nucleoside could only be determined in 32% of the urine samples.

Robust correlations were found between the mesor values of pseudouridine, 1-methyladenosine, 1-methylinosine, and adenosine, on the one hand, and between those of 1-methylguanosine, N2-N2-dimethylguanosine and 4-acetylcytidine on the other hand. Cytidine excretion appeared as independent from that of the other nucleosides.

Circadian and/or 12-h rhythms characterized the urinary excretion of pseudouridine for 48.3% of the patients (highest rate) and that of 4-acetylcytidine for 34.6% of the patients (lowest rate). Individual patients displayed marked rhythmic excretion patterns for up to seven nucleosides. Interestingly, the relative circadian amplitudes of nucleosides excretions were positively associated with the duration of rest for pseudouridine, adenosine, 4-acetylcytidine and cytidine, and with the dichotomy index I < O for 1-methylguanosine (Spearman correlation, p < 0.05). These results suggested that the rhythmic excretion patterns of modified nucleosides could reflect a host-mediated circadian control of cancer progression. Indeed, progression-free survival and overall survival were significantly improved in metastatic colorectal cancer patients whose I < O exceeded 97.5%^14^. This finding has been confirmed in other oncological clinical settings^36,37^.

A main limitation of our work involves the limited sample size in each St. Healthy controls would also be needed for establishing the physiological relations between host circadian biomarkers and the rhythmic turnover of nucleic acids in healthy tissues, as reflected in the urinary excretions of the modified nucleosides. A circadian rhythm was demonstrated in healthy adults for 8-hydroxy-2′-deoxyguanosine, with high values in the afternoon^33^, and for both pseudouridine and N2-N2-dimethylguanosine, with highest excretions occurring between 06:00 and 12:00^38^. The percent variations between average maximum value at acrophase and average minimum value at bathyphase (double amplitudes) were respectively 36%, 25.6%, and 12.7% of the mean for 8-hydroxy-2′-deoxyguanosine, pseudouridine and N2-N2-dimethylguanosine. In a circadian and sleep metabolomic study, sleep deprivation was associated with selective increase or decrease in 16 urinary metabolites^39^. This finding further supported the link between sleep duration and modified nucleosides excretion reported here.

Purine and pyrimidine synthesis and catabolism are rhythmically controlled by the circadian clock in mouse liver^40^. Clock gene Bmal1 silencing ablated these metabolic rhythms. Such Bmal1 silencing could result from host circadian disruption, as shown in mice on chronic jet lag^41^. Here, host circadian disruption was determined using 5-day time series of rest-activity monitoring. The median value of the dichotomy index I < O in our patient population was 97.9%, as compared to 97.5% for our prior cohorts of metastatic colorectal cancer patients, where this parameter was shown as an independent prognostic indicator of progression-free and overall survival^29^. I < O was here correlated with the circadian amplitude of 1-methylguanosine, and it was significantly inter-correlated with other rest-activity parameters that helped assess circadian timing system function in individual patients. The duration of rest over the 24 h was the host parameter, that displayed the strongest links with the rhythmic excretions of modified nucleosides, suggesting that the nucleic acids turnover pattern over the 24 h could be predominantly linked to the rest span. However, such correlations were not significant in the additional bootstrap uncertainty check, with 90th quantiles of Spearman correlation p-value > 0.1, a finding supporting the need for confirmation within a larger sample size. Interestingly, however, short sleep duration has been found to be associated with higher mortality in a large cohort of colorectal cancer survivors^42^.

In conclusion, we identified and ranked the main modified nucleosides that are excreted into the urines of patients with colorectal cancer metastases. Large interpatient variations were identified, that could reflect tumour or host nucleic acids turnover. Intrapatient changes in urinary nucleosides excretions over the 24-h occurred in nearly 1/3 to half of the cancer patients, consistently with the robustness of the circadian rhythm in rest-activity in these patients. The clinical relevance of urinary nucleosides excretions as biomarkers was further supported by the association of early changes in modified nucleosides on chemotherapy with disease progression and poor survival.

## Methods

### Study design

The patients had a histological proof of metastatic colorectal cancer, a performance status of 0 or 1 according to the classification of the World Health Organisation and adequate biology. They had received one, two or three prior chemotherapy protocols and provided a signed informed consent for their participation. Two studies (St) were implemented at the Department of Oncology in Paul Brousse University Hospital (Villejuif, France). The rest activity rhythm was monitored using a wrist-worn piezoelectric accelerometer watch (Mini-Motionlogger Actigraph, Ambulatory Monitoring, Ardsley, New-York, USA) for 5 days in St1 and for 17 days in St2 (data not reported here). The user-defined time interval for the count of wrist accelerations was 1 min. Each patient also completed a sleep and feeding diary questionnaire during the days of recording.

Both studies were sponsored by the National Institute for Health and Medical Research of France (INSERM), approved by the Ethics Committee of Kremlin Bicetre hospital (France) and registered with clinical trial numbers NCT01693835 and NCT01693861, respectively. They were conducted according to the “Ethics and methods for biological rhythm research on animals and human beings” and complied with the in force declaration of Helsinki principles^43^.

St1 investigated circadian changes in the urinary excretion of nine nucleosides according to patients’ characteristics and rest-activity rhythms. The wrist actigraph was worn continuously for 5 days, including 3 days of baseline, and 2 days during which urinary samples were self-collected (Fig. S3). The patients collected urine samples of 5–20 mL into pre-labelled sterile tubes, at 07:00 or upon awakening, then at 11:00, 15:00, 19:00 and 23:00 or just prior to retiring for two consecutive days, resulting in a total of 10 samples per patient.

St2 investigated changes in the urinary excretions of six nucleosides according to patients’ characteristics before, during and after a course of chrono-chemotherapy^44^ (Fig. S3). Early morning voids were collected daily for 3 days before and 14 days after the onset of a treatment course. Adverse events were rated after the chemotherapy course that was administered while on study, according to Common Terminology Criteria for Adverse Events (CTCAE) v4.0. Plasma CEA and CA19.9 were determined before and up to 2.5 months following inclusion. The survival of each patient was computed from the date of inclusion to the time of death or the last time known to be alive. These data were updated in March 2019.

### Urinary collections and nucleosides determinations

Nine modified nucleosides, namely pseudouridine, 1-methylguanosine, N2-N2-dimethylguanosine, 1-methyladenosine, 4-acetylcytidine, 1-methylinosine, adenosine, cytidine, and 8-hydroxy-2′-deoxyguanosine were determined with mass spectrometry. The quantification of urinary nucleosides was determined using the Q-Exactive, composed of the Orbitrap mass analyzer combined with a quadruple mass filter as front-end. This system was chosen because of its high sensitivity and the linearity performance of the High Resolution Mass Spectrometry (HRMS) based quantification^45^. Three of the nine nucleosides to be monitored were selected to be used as labeled internal standards for the entire quantification process (adenosine [ribose-13C5], 8-hydroxy-2′-deoxyguanosine (15N5) and 1-methyl-adenosine-D3). Due to the high concentration of most nucleosides in urine, the technique of “dilute and shoot” was chosen^46^. A dilution of the urine sample in water by a factor of 5 was applied as a pre-treatment overcoming the major matrix effects encountered in LC/MS bioanalysis techniques. Then, a 10 µL aliquot of the diluted urine was injected into the analytical system. The ultra-high pressure chromatographic (UHPLC) method was developed using a reverse phase column. The separation was performed on a column Acquity UPLC BEH Shield 1.7 µm 2.1 × 100 mm (Waters, France). Mobile phases were distributed in a gradient mode (Dionex Ultimate 3000 system). Detection by high-resolution mass spectrometry in full scan mode was accomplished using a Q-Exactive (Orbitrap technology, ThermoFisherScientifics) after ionization in positive mode. The total analysis runtime was 7 min. This method was successfully validated according to the European recommendations^47^.

### Statistical analysis

#### Hidden Markov Model (HMM) for quantifying of circadian rhythmicity in telemetric activity data

A recently developed harmonic Hidden Markov Model (HMM) approach^48^ was applied to compute numerical quantifiers associated with sleep quality and circadian rhythm in actigraphy data. The HMM is a well-known statistical model which assumes that the observed time series data are a realization of a Markov process with unobserved states. In this study, the HMM approach was applied to threshold the actigraphy measurement into three states in a probabilistic way, namely inactive/rest state (IA), moderately active (MA) state and highly active (HA) state. The following circadian rhythm associated parameters derived from HMM^34^ were p1-1 (the transition probability of staying in IA), which describles the probability of non-interrupted inactive/rest; median activity value at HA state; rest duration (integral of IA profile over the 24 h, i.e. dark grey area $a$ in Fig. S4; center of rest time (gravity center of IA, i.e. $c$ in Fig. S4); Rhythm Index, which assumes a value of 100% for a subject with regular daily bedtimes, an IA state with a transition probability of one (case in light grey, Fig. S4), and in contrast, a value of 0% in the absence of a circadian regulation of the rest duration (case in black, Fig. S4).

We also computed the autocorrelation at 24-h (r24), a measure of the regularity of the 24-h rest-activity pattern from day to day, as well as the dichotomy index (I < O), defined as the percentage of activity in-bed (I) that was less than the median activity out-of-bed (O)^28,49^. Furthermore, we also computed for each patient the dominant period using the spectrum-resampling (SR) algorithm of Costa et al.^50^, as well as the acrophase and amplitude using a cosinor model.

#### Cosinor analysis for quantifying of circadian rhythmicity in urinary nucleosides excretion

A multiple-component cosinor model^51^ was applied to describe the circadian pattern of nucleosides excretion for St 1. More specifically the model contained two cosine functions with respective periods ${T}_{12}=12$-h and ${T}_{24}=24$-h:

1 $$y\left(t\right)=M+{A}_{12}\mathrm{cos}\left(\frac{2\pi t}{{T}_{12}}+{\theta }_{12}\right)+{A}_{24}\mathrm{cos}\left(\frac{2\pi t}{{T}_{24}}+{\theta }_{24}\right)+e\left(t\right),$$

where $y\left(t\right)$ is the urinary nucleosides excretion at time *t*; $M$ is the mesor (mean level); ${A}_{12},{A}_{24}$ and ${\theta }_{12},{\theta }_{24}$ are the corresponding amplitude and acrophase for each cosine term; $e\left(t\right)$ is the error.

The five parameters, i.e., $M$, ${A}_{12},{A}_{24}$, ${\theta }_{12},{\theta }_{24}$ in the non-linear model (1), were estimated using the *R* package *nlme*^52^ To avoid over-fitting, individuals with less than seven timed nucleosides excretion samples were excluded from the cosinor analysis. We report the overall circadian amplitude $A$, i.e. half difference of the highest and lowest values of curve fitting $\widehat{y}\left(t\right)$, relative amplitude (% of mesor) and the overall acrophase $\theta$, i.e. acrophase of $\widehat{y}\left(t\right)$. Nucleosides excretions were identified to have significant rhythmic pattern at 24-h if either amplitude (${A}_{12}$ or ${A}_{24}$) was significantly different from zero (*t*-test with p-value $\le$ 0.1). Note that the liberal 0.1 p-value threshold was selected due to limited sample size, i.e. six parameters in model (1) need to be estimated by up to 10 data samples. In order to improve upon the model fit and the assumption of Gaussianity for the residuals, the log transformation was applied to the raw nucleosides excretion before the cosinor analysis. The mesor and amplitude results were transformed back to the original scale for reporting.

#### Correlation between circadian parameter estimates

The Spearman rank correlation was computed between any pair of parameters related to rest-activity and urinary nucleosides excretion patterns. Since large uncertainties could be expected for all circadian parameter estimates related to nucleosides excretion, correlations related with nucleosides circadian parameters were further verified by bootstrap uncertainty estimates^53^. One thousand bootstrap trials were generated via sampling the residuals in Eq. (1) with replacement and 1000 bootstrap nucleosides paramters were computed. The correlations were identified to be significant if the 90% quantiles of bootstrap Spearman correlation p-values $\le$ 0.1.

#### Regression analysis of parameter estimates versus patients’ characteristics

Multivariate regression analysis was applied to estimate the relation between main patient characteristics and response variables. The latter included the estimated parameters characterizing the rest-activity cycle, namely the dichotomy index I < O, the estimated probability of staying in the rest state p1-1, the rest duration, the Rhythm Index RI, the median value of the HA state and the autocorrelation r24; and those in the nucleosides urinary excretion, such as the mesor estimates, the relative amplitude (% of mesor) and the number of nucleosides with significant rhythmicity pattern at 24-h. Stepwise model selection based on Akaike’s information criterion^54^, as implemented in the *R* function *stepAIC, *was applied to obtain an appropriate model for each response variable.

## Supplementary Information

Supplementary Information.
