# Supplementary material for: Circadian and chemotherapy-related changes in urinary modified nucleosides excretion in patients with metastatic colorectal cancer
Source: Sci Rep. 2021 Dec 14;11:24015. doi: 10.1038/s41598-021-03247-2 (PMC8671418; doi:10.1038/s41598-021-03247-2)
Supplement: Supplementary file 1 — Supplementary Information. [file 41598_2021_3247_MOESM1_ESM.docx]

**Circadian and chemotherapy-related changes in urinary modified nucleosides excretion in patients with metastatic colorectal cancer**

**Running tittle: Urinary nucleosides dynamics in colorectal cancer**

**Dulong S^1^,^2^*, Huang Q^,3, 4^*, Innominato P F^3, 5^, Karaboue A ^1, 7^, Bouchahda M^1, 6, 7^, Pruvost A^8, 9^, Théodoro F^8, 9^, Agrofoglio L A^10^, Adam R^1,2,11^, Finkenstädt B^4^, and Lévi F ^1,3,6^**

**Supplementary Information**

**Table S1: Distribution of rest-activity parameters (St 1).** Parameters are summarised by their mean values, median values, 1^st^-3^rd^ quartiles and range using imputed values from each individual patient.

|  | **Parameters** | | | |
| --- | --- | --- | --- | --- |
| **Rest-activity indices** | **Mean** | **Median** | **1^st^-3^rd^ quartiles** | **Range** |
| Dichotomy Index I<O (%) | 96.5 | 97.9 | 95.9 – 98.5 | 85.0 – 99.7 |
| Rhythm Index (%) | 67.1 | 66.7 | 59.5 – 74.4 | 41.9 – 91.2 |
| Transition probability of staying in inactive/rest state (p1-1) | 0.944 | 0.954 | 0.944 – 0.962 | 0.810 - 0.979 |
| Autocorrelation r24 | 0.347 | 0.379 | 0.247 – 0.425 | 0.095 – 0.654 |
| Median value of highly active state (accelerometers per min) | 236 | 235 | 223 - 269 | 102 - 296 |
| Daily rest duration (hour and min) | 9h34min | 9h25m | 8h14min – 10h43min | 7h16min-12h31min |
| Center rest time (clock time) | 03:20 | 03:25 | 03: 10 – 03:50 | 23:20 – 04:35 |
| *Spectral analyses* |  | | | |
| Dominant period (hour and min) | 23h54min | 23h48min | 23h48min-24h00min | 22h42min-25h00min |
| *24h parameters (Cosinor)* |  | | | |
| Mesor ($u$g/g creatinine) | 101.75 | 105 | 89-117.25 | 34-143 |
| Amplitude ($u$g/g creatinine) | 84.46 | 86 | 75.75-101 | 19-125 |
| Acrophase (clock time) | 14:39 | 14:38 | 13:59-15:24 | 11:46-16:41 |

**
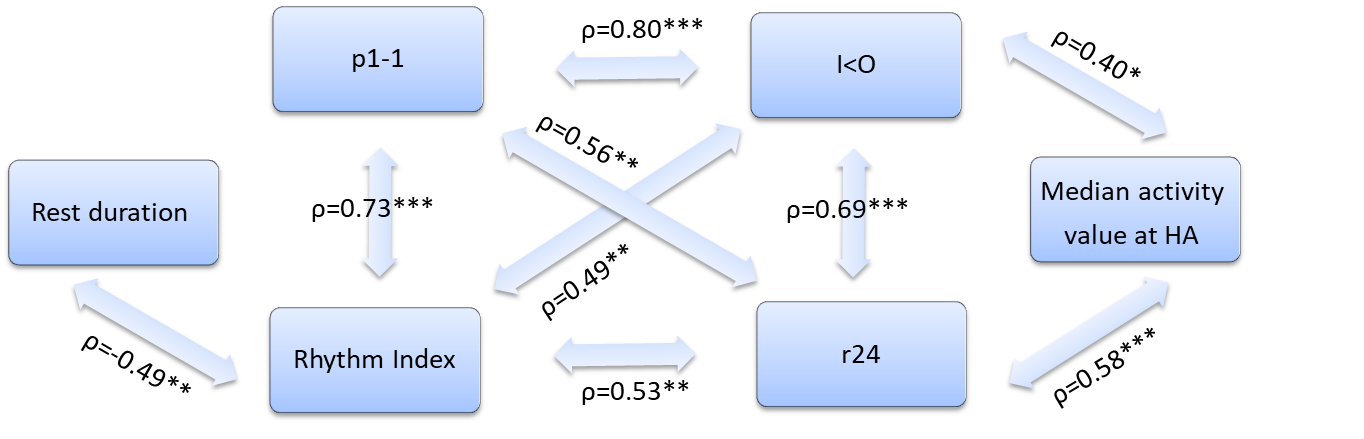
Figure S1: Diagram of intercorrelations between rest-activity parameters (St 1).**

Spearman’s rank correlation coefficients ρ with stars indicating p-value levels, i.e. 0 ‘***’ 0.001 ‘**’ 0.01 ‘*’ 0.05 ‘^’ 0.1.

**(3)**

**Figure S2: Examples of inter-subject differences in trends of cytidine excretions before, during and after chronochemotherapy in three patients (St 2).**

Urinary nucleoside excretion measurements are shown as dots in both upper panels, with no statistically significant trend **(1)** for the data in the upper left panel, and significantly increasing or decreasing trends **(2)** plotted as continuous lines for the data in the upper right panel (p-value $\leq$ 0.05). **(3)** The lower panel depicts the Kaplan Meir survival curves for the 6 patients with a trend in at least one nucleoside, and for those 8 patients with stable urinary nucleoside excretions (no trend).

**
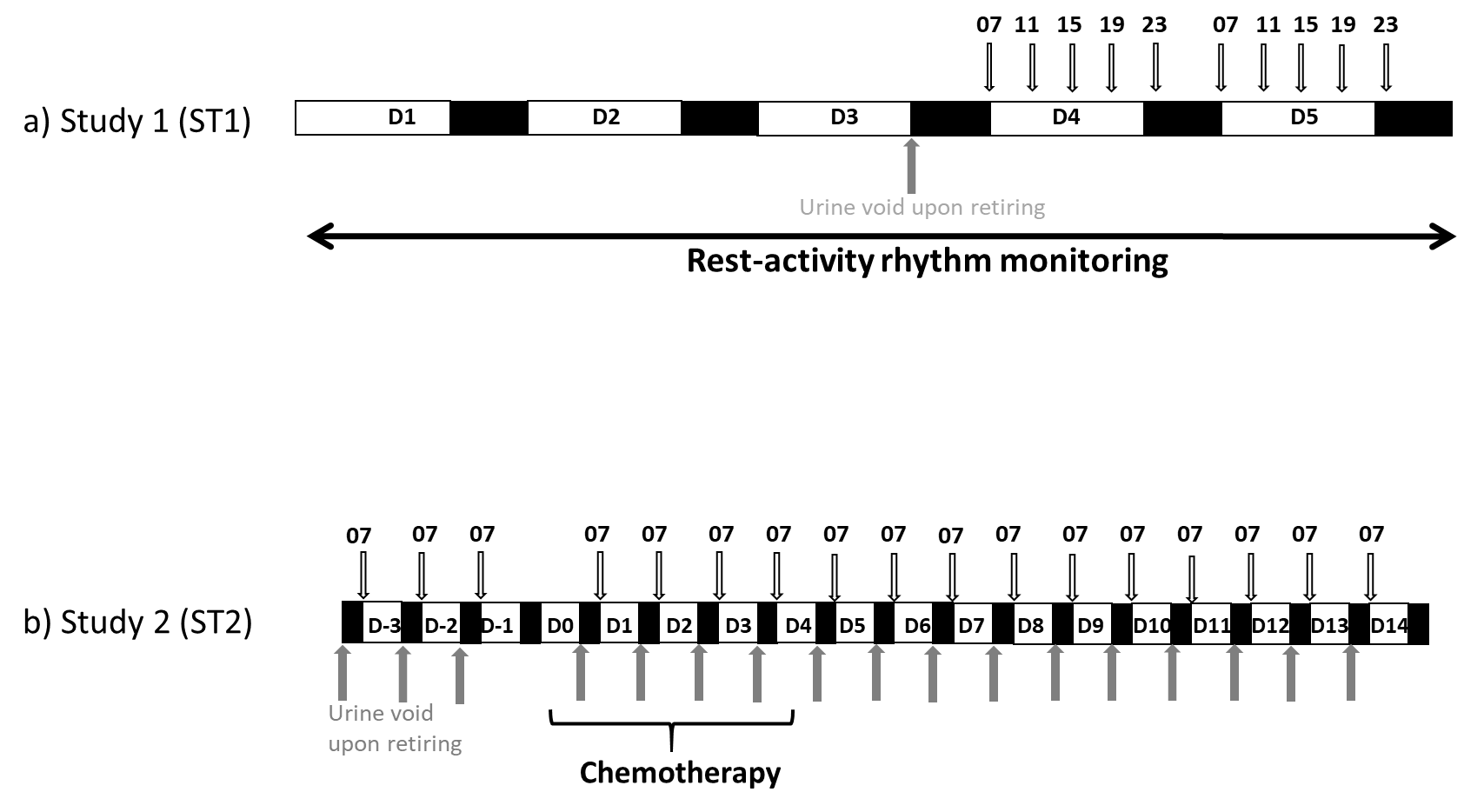
**

**Figure S3: Study designs** Time frames for urinary samples collection (vertical arrows) and wrist actigraphy in Study 1 (St1), and urinary samples collection in Study 2 (St2).

**Figure S4: Diagram for deriving Rhythm Index.**

Dark grey area gives example probability of IA state with integral $a$ and gravity center $c$. The light grey rectangular area corresponds to rest perfectly round the $c$ with probability one for which Rhythm Index value of 100%. The black rectangular area corresponds to rest in the absence of circadian rhythm with Rhythm Index value of 0%. Note that the integral size of the three profiles, i.e. black, dark grey and light grey, are all correspond to the rest duration $a$ *(Huang et al. 2018)*.
